# Supplementary material for: How not to be seen: predicting unseen enzyme functions using contrastive learning
Source: Bioinformatics. 2026 Jul 7;42(Suppl 1):btag215. doi: 10.1093/bioinformatics/btag215 (PMC13340170; doi:10.1093/bioinformatics/btag215)
Supplement: btag215_Supplementary_Data [file btag215_supplementary_data.pdf]

SUPPLEMENTARY MATERIAL

Supplementary Information: How Not to be Seen: Predicting Unseen Enzyme Functions using Contrastive Learning

Xiang Ma[1,3,*], Parnal Joshi[2], Iddo Friedberg[2], and Qi Li[1,*]

[1]Computer Science and [2]Veterinary Microbiology and Preventive Medicine, Iowa State University, 50014, IA, USA
[3]Biochemistry, Grand View University, 50316, IA, USA

* Corresponding author: qli@iastate.edu

Table S1. Detailed metrics for unseen evaluations. Mean ± half-width of the 95% bootstrap confidence interval for EC1–EC3 metrics on `Unseen Test` and the low-similarity subsets (`Unseen Test 50%`, `Unseen Test 30%`, `Unseen Test 10%`).

Model	Test set	EC1 Acc.	EC1 F1	EC1 Prec.	EC1 Rec.	EC2 Acc.	EC2 F1	EC2 Prec.	EC2 Rec.	EC3 Acc.	EC3 F1	EC3 Prec.	EC3 Rec.
BLAST	Unseen Test	0.4894 ± 0.0096	0.3563 ± 0.0107	0.3586 ± 0.0101	0.4075 ± 0.0131	0.3112 ± 0.0091	0.2143 ± 0.0163	0.2270 ± 0.0190	0.2541 ± 0.0191	0.2524 ± 0.0085	0.1355 ± 0.0105	0.1621 ± 0.0136	0.1708 ± 0.0143
ProteInfer	Unseen Test	0.3983 ± 0.0091	0.2956 ± 0.0102	0.2879 ± 0.0097	0.3461 ± 0.0122	0.1943 ± 0.0078	0.1156 ± 0.0108	0.1529 ± 0.0186	0.1405 ± 0.0159	0.1627 ± 0.0073	0.0779 ± 0.0064	0.1160 ± 0.0119	0.0986 ± 0.0108
GloEC	Unseen Test	0.6248 ± 0.0096	0.5539 ± 0.0140	0.5651 ± 0.0148	0.5581 ± 0.0155	0.3619 ± 0.0098	0.2274 ± 0.0137	0.2641 ± 0.0215	0.2691 ± 0.0215	0.2716 ± 0.0086	0.1312 ± 0.0082	0.1640 ± 0.0120	0.1781 ± 0.0146
CLEAN	Unseen Test	0.5950 ± 0.0090	0.5257 ± 0.0142	0.5497 ± 0.0155	0.5323 ± 0.0158	0.3854 ± 0.0099	0.2261 ± 0.0137	0.2714 ± 0.0175	0.2739 ± 0.0239	0.3144 ± 0.0092	0.1498 ± 0.0097	0.1945 ± 0.0112	0.1841 ± 0.0145
Raw ESM	Unseen Test	0.5519 ± 0.0053	0.4982 ± 0.0063	0.5037 ± 0.0070	0.4989 ± 0.0066	0.3210 ± 0.0049	0.1817 ± 0.0091	0.2247 ± 0.0133	0.2147 ± 0.0156	0.2500 ± 0.0046	0.1215 ± 0.0043	0.1713 ± 0.0078	0.1476 ± 0.0085
EnzPlacer	Unseen Test	0.6387 ± 0.0093	0.5651 ± 0.0128	0.5539 ± 0.0141	0.5961 ± 0.0143	0.4350 ± 0.0099	0.2614 ± 0.0157	0.2804 ± 0.0177	0.3095 ± 0.0237	0.3563 ± 0.0096	0.1678 ± 0.0098	0.1982 ± 0.0123	0.2133 ± 0.0153
BLAST	Unseen Test 50%	0.4807 ± 0.0103	0.3496 ± 0.0126	0.3527 ± 0.0122	0.4005 ± 0.0160	0.2997 ± 0.0091	0.2028 ± 0.0167	0.2191 ± 0.0172	0.2407 ± 0.0200	0.2402 ± 0.0084	0.1263 ± 0.0106	0.1515 ± 0.0132	0.1596 ± 0.0147
ProteInfer	Unseen Test 50%	0.4091 ± 0.0052	0.3146 ± 0.0048	0.3040 ± 0.0047	0.3463 ± 0.0057	0.1944 ± 0.0041	0.1123 ± 0.0055	0.1430 ± 0.0102	0.1314 ± 0.0078	0.1624 ± 0.0037	0.0724 ± 0.0042	0.1066 ± 0.0080	0.0852 ± 0.0058
GloEC	Unseen Test 50%	0.6189 ± 0.0096	0.5467 ± 0.0143	0.5590 ± 0.0162	0.5512 ± 0.0153	0.3522 ± 0.0097	0.2130 ± 0.0124	0.2507 ± 0.0204	0.2578 ± 0.0207	0.2601 ± 0.0088	0.1212 ± 0.0085	0.1548 ± 0.0112	0.1548 ± 0.0112
CLEAN	Unseen Test 50%	0.5884 ± 0.0096	0.5190 ± 0.0150	0.5442 ± 0.0159	0.5253 ± 0.0161	0.3764 ± 0.0093	0.2157 ± 0.0130	0.2605 ± 0.0157	0.2634 ± 0.0233	0.3044 ± 0.0090	0.1406 ± 0.0106	0.1828 ± 0.0119	0.1746 ± 0.0153
Raw ESM	Unseen Test 50%	0.5107 ± 0.0092	0.4327 ± 0.0145	0.4357 ± 0.0153	0.4476 ± 0.0159	0.2794 ± 0.0092	0.1736 ± 0.0120	0.2274 ± 0.0184	0.2011 ± 0.0198	0.2067 ± 0.0080	0.1054 ± 0.0075	0.1589 ± 0.0116	0.1268 ± 0.0133
EnzPlacer	Unseen Test 50%	0.6329 ± 0.0093	0.5587 ± 0.0128	0.5475 ± 0.0142	0.5905 ± 0.0149	0.4267 ± 0.0098	0.2512 ± 0.0148	0.2694 ± 0.0144	0.2999 ± 0.0246	0.3469 ± 0.0095	0.1597 ± 0.0108	0.1873 ± 0.0122	0.2052 ± 0.0156
BLAST	Unseen Test 30%	0.4158 ± 0.0114	0.2727 ± 0.0266	0.2857 ± 0.0266	0.3126 ± 0.0328	0.2261 ± 0.0093	0.1326 ± 0.0163	0.1558 ± 0.0211	0.1607 ± 0.0226	0.1726 ± 0.0081	0.0754 ± 0.0093	0.0979 ± 0.0134	0.0982 ± 0.0130
ProteInfer	Unseen Test 30%	0.3471 ± 0.0057	0.2283 ± 0.0056	0.2223 ± 0.0050	0.2640 ± 0.0089	0.1360 ± 0.0041	0.0730 ± 0.0064	0.1073 ± 0.0112	0.0881 ± 0.0096	0.1043 ± 0.0036	0.0396 ± 0.0027	0.0630 ± 0.0067	0.0513 ± 0.0058
GloEC	Unseen Test 30%	0.5783 ± 0.0106	0.5000 ± 0.0196	0.4969 ± 0.0198	0.4876 ± 0.0179	0.2945 ± 0.0096	0.1605 ± 0.0108	0.1935 ± 0.0167	0.2028 ± 0.0217	0.2045 ± 0.0090	0.0849 ± 0.0079	0.1147 ± 0.0108	0.1148 ± 0.0140
CLEAN	Unseen Test 30%	0.5398 ± 0.0113	0.4516 ± 0.0161	0.4902 ± 0.0167	0.4553 ± 0.0205	0.3183 ± 0.0099	0.1687 ± 0.0114	0.2119 ± 0.0146	0.2031 ± 0.0212	0.2497 ± 0.0093	0.0983 ± 0.0075	0.1392 ± 0.0111	0.1281 ± 0.0134
Raw ESM	Unseen Test 30%	0.4682 ± 0.0110	0.3745 ± 0.0166	0.3761 ± 0.0179	0.3998 ± 0.0203	0.2278 ± 0.0094	0.1216 ± 0.0097	0.1696 ± 0.0167	0.1481 ± 0.0193	0.1612 ± 0.0081	0.0691 ± 0.0067	0.1134 ± 0.0116	0.0825 ± 0.0112
EnzPlacer	Unseen Test 30%	0.5919 ± 0.0108	0.4971 ± 0.0174	0.4861 ± 0.0171	0.5357 ± 0.0203	0.3765 ± 0.0106	0.2016 ± 0.0135	0.2206 ± 0.0192	0.2505 ± 0.0264	0.2994 ± 0.0098	0.1238 ± 0.0091	0.1483 ± 0.0119	0.1670 ± 0.0148
BLAST	Unseen Test 10%	0.3239 ± 0.0121	0.1821 ± 0.0205	0.2033 ± 0.0217	0.2042 ± 0.0271	0.1255 ± 0.0089	0.0483 ± 0.0086	0.0644 ± 0.0146	0.0572 ± 0.0136	0.0818 ± 0.0075	0.0280 ± 0.0046	0.0398 ± 0.0098	0.0367 ± 0.0082
ProteInfer	Unseen Test 10%	0.3251 ± 0.0078	0.1804 ± 0.0061	0.1826 ± 0.0053	0.2044 ± 0.0105	0.1023 ± 0.0046	0.0348 ± 0.0029	0.0446 ± 0.0041	0.0465 ± 0.0052	0.0738 ± 0.0040	0.0195 ± 0.0020	0.0273 ± 0.0058	0.0280 ± 0.0038
GloEC	Unseen Test 10%	0.5326 ± 0.0142	0.3970 ± 0.0260	0.4092 ± 0.0267	0.4082 ± 0.0294	0.2344 ± 0.0117	0.1183 ± 0.0103	0.1449 ± 0.0173	0.1500 ± 0.0239	0.1550 ± 0.0100	0.0634 ± 0.0087	0.0873 ± 0.0127	0.0823 ± 0.0121
CLEAN	Unseen Test 10%	0.4689 ± 0.0135	0.3502 ± 0.0222	0.4073 ± 0.0299	0.3581 ± 0.0281	0.2468 ± 0.0122	0.1209 ± 0.0130	0.1704 ± 0.0180	0.1614 ± 0.0270	0.1813 ± 0.0102	0.0662 ± 0.0089	0.1033 ± 0.0123	0.0866 ± 0.0121
Raw ESM	Unseen Test 10%	0.4252 ± 0.0141	0.2921 ± 0.0195	0.2976 ± 0.0193	0.3224 ± 0.0281	0.1715 ± 0.0102	0.0791 ± 0.0092	0.1161 ± 0.0176	0.0931 ± 0.0168	0.1188 ± 0.0092	0.0464 ± 0.0067	0.0765 ± 0.0108	0.0585 ± 0.0101
EnzPlacer	Unseen Test 10%	0.5270 ± 0.0139	0.3859 ± 0.0215	0.3782 ± 0.0199	0.4245 ± 0.0313	0.3034 ± 0.0130	0.1547 ± 0.0153	0.1775 ± 0.0198	0.1857 ± 0.0232	0.2260 ± 0.0112	0.0899 ± 0.0091	0.1130 ± 0.0111	0.1166 ± 0.0126

Table S2. Detailed metrics for seen evaluations. Mean ± half-width of the 95% bootstrap confidence interval for EC4 prediction performance (Accuracy, macro-F1, Precision, Recall) and hierarchical accuracies (EC1–EC3) on `Seen Test 50%`, `Seen Test 30%`, and `Seen Test 10%`.

Model	Test set	EC4 Acc.	EC4 F1	EC4 Prec.	EC4 Rec.	EC1 Acc.	EC2 Acc.	EC3 Acc.
BLAST	Seen Test 50%	0.8971 ± 0.0071	0.7342 ± 0.0138	0.7541 ± 0.0137	0.7276 ± 0.0142	0.9651 ± 0.0047	0.9609 ± 0.0050	0.9571 ± 0.0052
ProteInfer	Seen Test 50%	0.7985 ± 0.0101	0.7270 ± 0.0124	0.7500 ± 0.0128	0.7210 ± 0.0122	0.8546 ± 0.0089	0.8378 ± 0.0087	0.8298 ± 0.0094
GloEC	Seen Test 50%	0.6557 ± 0.0127	0.4788 ± 0.0132	0.4914 ± 0.0141	0.5107 ± 0.0130	0.9270 ± 0.0067	0.8462 ± 0.0091	0.8028 ± 0.0103
CLEAN	Seen Test 50%	0.8775 ± 0.0084	0.7262 ± 0.0141	0.7363 ± 0.0141	0.7350 ± 0.0146	0.9772 ± 0.0039	0.9615 ± 0.0051	0.9474 ± 0.0056
Raw ESM	Seen Test 50%	0.8544 ± 0.0085	0.7135 ± 0.0144	0.7225 ± 0.0145	0.7215 ± 0.0147	0.9741 ± 0.0041	0.9594 ± 0.0050	0.9410 ± 0.0056
EnzPlacer	Seen Test 50%	0.9098 ± 0.0074	0.7529 ± 0.0144	0.7601 ± 0.0147	0.7590 ± 0.0144	0.9827 ± 0.0033	0.9733 ± 0.0041	0.9658 ± 0.0046
BLAST	Seen Test 30%	0.5820 ± 0.0413	0.4185 ± 0.0384	0.4392 ± 0.0422	0.4101 ± 0.0393	0.6744 ± 0.0402	0.6660 ± 0.0412	0.6554 ± 0.0402
ProteInfer	Seen Test 30%	0.6561 ± 0.0369	0.5940 ± 0.0373	0.6150 ± 0.0373	0.5853 ± 0.0377	0.7247 ± 0.0369	0.7041 ± 0.0369	0.6921 ± 0.0369
GloEC	Seen Test 30%	0.3516 ± 0.0377	0.1933 ± 0.0274	0.2024 ± 0.0277	0.1967 ± 0.0280	0.8070 ± 0.0334	0.6492 ± 0.0386	0.6063 ± 0.0403
CLEAN	Seen Test 30%	0.6326 ± 0.0455	0.3765 ± 0.0390	0.3838 ± 0.0392	0.3815 ± 0.0384	0.9027 ± 0.0264	0.8397 ± 0.0339	0.7913 ± 0.0359
Raw ESM	Seen Test 30%	0.6113 ± 0.0444	0.3545 ± 0.0359	0.3610 ± 0.0363	0.3602 ± 0.0375	0.8806 ± 0.0265	0.8083 ± 0.0349	0.7632 ± 0.0370
EnzPlacer	Seen Test 30%	0.6836 ± 0.0423	0.4016 ± 0.0394	0.4077 ± 0.0400	0.4073 ± 0.0400	0.9110 ± 0.0264	0.8587 ± 0.0328	0.8208 ± 0.0359
BLAST	Seen Test 10%	0.5608 ± 0.0665	0.4321 ± 0.0642	0.4594 ± 0.0659	0.4227 ± 0.0643	0.6305 ± 0.0616	0.6207 ± 0.0665	0.6158 ± 0.0665
ProteInfer	Seen Test 10%	0.6446 ± 0.0575	0.5933 ± 0.0552	0.6170 ± 0.0572	0.5826 ± 0.0547	0.7106 ± 0.0596	0.6915 ± 0.0617	0.6872 ± 0.0574
GloEC	Seen Test 10%	0.3851 ± 0.0618	0.2056 ± 0.0430	0.2125 ± 0.0439	0.2103 ± 0.0449	0.7873 ± 0.0554	0.6106 ± 0.0660	0.5851 ± 0.0617
CLEAN	Seen Test 10%	0.6371 ± 0.0616	0.3692 ± 0.0617	0.3809 ± 0.0625	0.3683 ± 0.0618	0.9068 ± 0.0395	0.8280 ± 0.0517	0.7692 ± 0.0591
Raw ESM	Seen Test 10%	0.6176 ± 0.0640	0.3404 ± 0.0565	0.3549 ± 0.0585	0.3386 ± 0.0572	0.8663 ± 0.0468	0.7688 ± 0.0567	0.7538 ± 0.0567
EnzPlacer	Seen Test 10%	0.6757 ± 0.0640	0.3763 ± 0.0613	0.3866 ± 0.0630	0.3749 ± 0.0614	0.9061 ± 0.0419	0.8469 ± 0.0493	0.8073 ± 0.0542

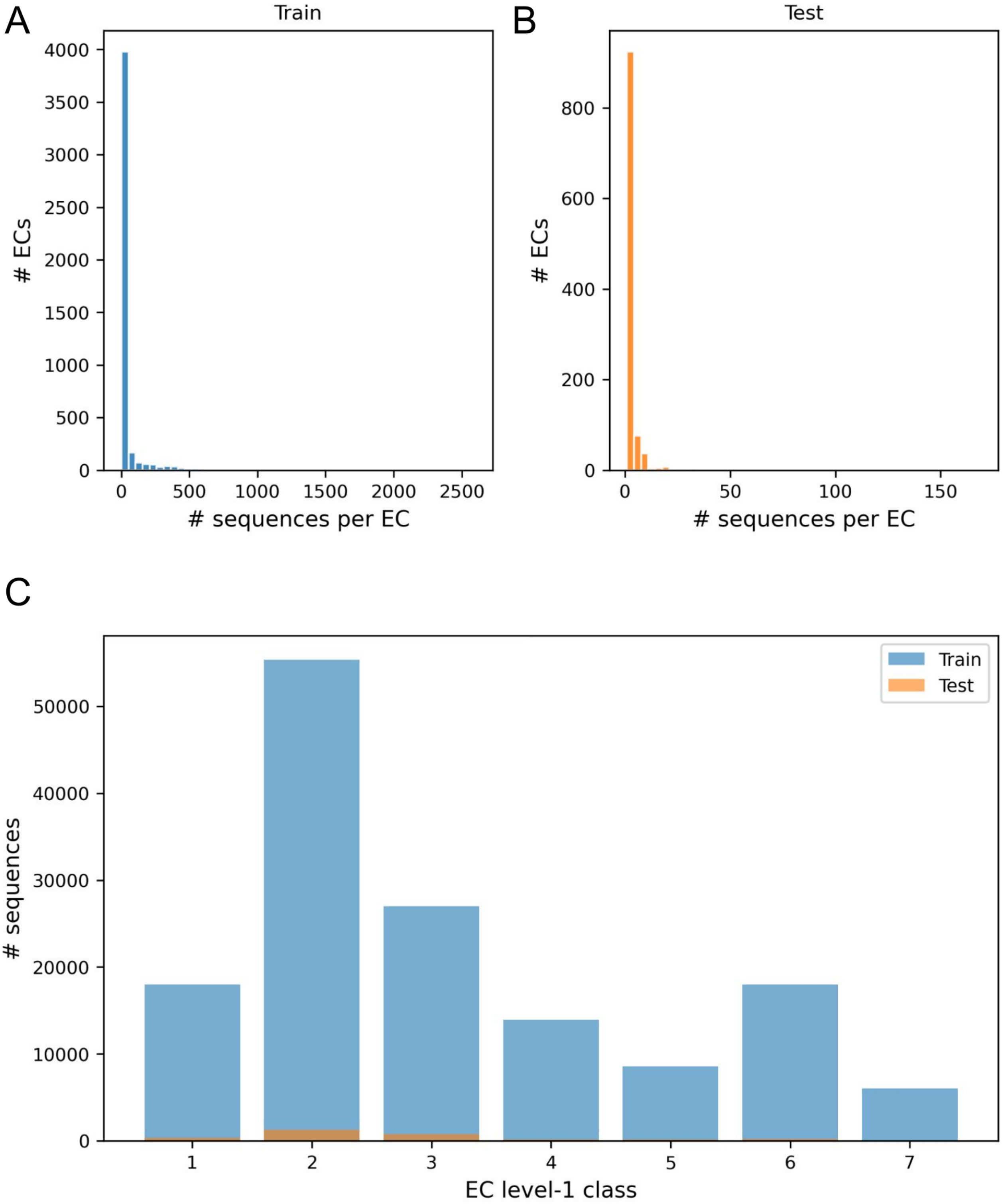

Supplementary Figure S1. Seen dataset statistics. (A–B) Distributions of the number of sequences per EC in the training and test splits. (C) Sequence counts per EC level-1 class for train and test.

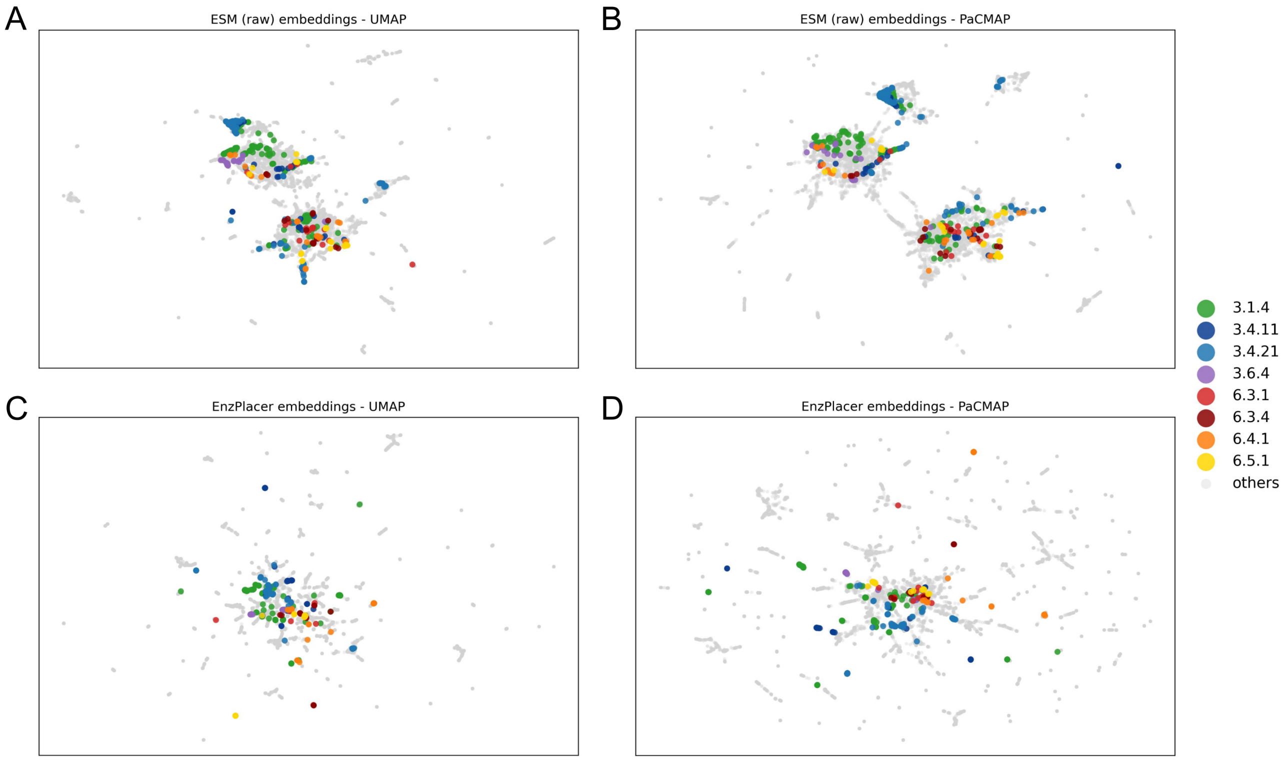

Supplementary Figure S2. UMAP and PaCMAP visualizations of raw ESM and EnzPlacer embeddings on the unseen test set. (A) UMAP projection of raw ESM embeddings. (B) PaCMAP projection of raw ESM embeddings. (C) UMAP projection of EnzPlacer embeddings. (D) PaCMAP projection of EnzPlacer embeddings. Highlighted colors indicate representative EC classes, while gray points denote all other classes.
